# Glycation Increases the Risk of Microbial Traversal through an Endothelial Model of the Human Blood-Brain Barrier after Use of Anesthetics

**DOI:** 10.3390/jcm9113672

**Published:** 2020-11-16

**Authors:** Veronika Weber, Heidi Olzscha, Timo Längrich, Carla Hartmann, Matthias Jung, Britt Hofmann, Rüdiger Horstkorte, Kaya Bork

**Affiliations:** 1 Institut für Physiologische Chemie, Martin-Luther-Universität Halle-Wittenberg, Hollystr. 1, 06114 Halle (Saale), Germany; veronika.weber@uk-halle.de (V.W.); timo.laengrich@uk-halle.de (T.L.); ruediger.horstkorte@uk-halle.de (R.H.); kaya.bork@uk-halle.de (K.B.); 2 Klinik und Poliklinik für Psychiatrie, Psychotherapie und Psychosomatik, Martin-Luther-Universität Halle-Wittenberg, Julius-Kühn-Str. 7, 06112 Halle (Saale), Germany; carla.hartmann@uk-halle.de (C.H.); matthias.jung@uk-halle.de (M.J.); 3 Klinik und Poliklinik für Herzchirurgie, Universitätsklinikum Halle (Saale), Ernst-Grube-Str. 20, 06120 Halle (Saale), Germany; britt.hofmann@uk-halle.de

**Keywords:** advanced glycation endproducts (AGE), anesthetics, ascorbic acid, blood–brain barrier, diabetes mellitus, glycation, human brain microvascular endothelial cells, meningitis, microbial traversal, propofol

## Abstract

The function of the human blood–brain barrier (BBB), consisting mainly of the basement membrane and microvascular endothelial cells, is to protect the brain and regulate its metabolism. Dysfunction of the BBB can lead to increased permeability, which can be linked with several pathologies, including meningitis, sepsis, and postoperative delirium. Advanced glycation end products (AGE) are non-enzymatic, posttranslational modifications of proteins, which can affect their function. Increased AGE levels are strongly associated with ageing and degenerative diseases including diabetes. Several studies demonstrated that the formation of AGE interfere with the function of the BBB and may change its permeability for soluble compounds. However, it is still unclear whether AGE can facilitate microbial traversal through the BBB and how small compounds including anesthetics modulate this process. Therefore, we developed a cellular model, which allows for the convenient testing of different factors and compounds with a direct correlation to bacterial traversal through the BBB. Our results demonstrate that both glycation and anesthetics interfere with the function of the BBB and promote microbial traversal. Importantly, we also show that the essential nutrient and antioxidant ascorbic acid, commonly known as vitamin C, can reduce the microbial traversal through the BBB and partly reverse the effects of AGE.

## 1. Introduction

Due to the increased life span, age-related diseases, and consecutive demographic changes, health and mental performance in the elderly is becoming more important. The involvement of advanced glycation end products (AGE) is one possible cause for molecular ageing and correlates with the development of age-related diseases, in particular diabetes [1]. During normal ageing, AGE accumulate in the organism, thus contributing to the molecular mechanisms of cellular ageing [2,3]. AGE are built during glycation, a non-enzymatically condensation reaction between the carbonyl group of reducing carbohydrates or metabolites and free amino groups. The reaction includes the reversible formation of Schiff bases, which further rearrange to so-called Amadori products, and which are finally converted to AGE [4]. Histopathologic studies already showed accumulations of a diversity of AGE in the lung, liver, kidney, or amyloid plaques in Alzheimer’s disease [5]. Fragmentation of Amadori products can result in the formation of methylglyoxal (MGO), which is also formed as a regular by-product of glycolysis. Up to 0.4% of all glucose molecules are metabolized to MGO per cycle of glycolysis, whereby concentrations can be even higher in impaired glucose utilization or hyperglycemia [6,7]. MGO is a highly reactive dicarbonyl molecule, leading to the formation of different AGE. In general, the accumulation of dicarbonyl components is also referred to as carbonyl stress [2,8].

The pathology of AGE is mediated through different mechanisms. The generation of protein modifications or cross-links of extra- or intracellular components can alter protein function or even results in a complete loss of protein function [1]. Another aspect to be mentioned is the binding of AGE to receptors, such as the receptor for advanced glycation end products (RAGE), thereby activating pro-inflammatory signal transduction cascades [8]. Endothelial cells cultivated in high glucose concentrations show an increased RAGE activity which leads to increased endothelial cell permeability [9].

Since AGE can basically affect all proteins, they also interfere with proteins of the blood-brain barrier (BBB). The BBB is a tight barrier which separates the blood from the brain, regulates the access of large and hydrophilic molecules to the brain and ensures that the brain can act in a metabolically strictly controlled compartment [10]. The most important cell type forming the tight BBB are the brain microvascular endothelial cells (BMECs). They have some unique features which distinguish them from other endothelial cells, such as a high number of tight junctions and adherens junctions [11] and a continuous basement membrane [12]. There are certain marker proteins for the tight junctions between the BMECs, e.g., occludin, whose level are high in intact BMECs [13]. Another marker protein in BMECs is VE-cadherin, a transmembrane protein which mainly constitutes the adherens junctions and is linked to the actin cytoskeleton with catenins [14]. Many diseases are associated with increased permeability of the BBB, including bacterial meningitis and sepsis [15]. Additionally, iatrogenic disorders can be accompanied with a more permeable BBB, an example would be the postoperative delirium, which is strongly associated with preoperative infections [16,17] and diabetes has been found as independent predisposing factor [18].

Multiple studies have shown that patients with diabetes have a two-fold higher risk of developing meningitis or encephalitis with hyperglycemia remaining a risk factor for severe outcomes and increase of mortality [19,20,21]. In addition to the immunosuppressing effects of diabetes, there are indications that AGE can be causative for an increased permeability of the BBB and some mechanistic aspects give further evidence for this hypothesis. For instance, Shimizu et al. showed in a study that AGE reduced the expression of claudin 5 in BMECs by increasing autocrine signaling via the vascular endothelial growth factor (VEGF) cascade. They also proposed that AGE increase the degree of autocrine TGF-β signaling by pericytes, and thereby weaken the BBB through the up-regulation of VEGF and MMP-2 in BMECs under diabetic conditions [22]. The expression of the proteins zonula occludens-1 (ZO-1) and occludin is downregulated by MGO in a model built with THBMEC [23]. Another study revealed that AGE can cause phosphorylation of myosin in murine brain microvascular endothelial cells with p38 and Rho kinase pathway activation [24]. Kim et al. showed that the primary entry site of circulating bacteria is the microvessels [25]. However, the BBB prevents the entry of bacteria. The ability of bacteria entering the brain via transcellular as well as paracellular traversal is related to the interaction with proteins which are also affected by AGE, such as VEGF [26,27], ZO-1, or occludin [28].

However, it has not been clarified, so far, how and to what degree AGE can cause an increased permeability of the BBB that even bacteria could traverse easier through the barrier, leading to the mentioned disease of bacterial meningitis. It has also not been systematically evaluated, how compounds affecting the brain, especially anesthetics, can influence the permeability of the BBB. There are studies showing that propofol as a common anesthetic induce cellular stress and support the breakdown of BBB [29,30]. In addition, some studies proof the neuroprotective effect of propofol by tighten the BBB and lower incidence of post-operative delirium compared with other anesthetics such as sevoflurane [31,32].

In this study, we report the usage of a newly developed BBB model system [33] to analyze the effects of AGE on the permeability of the BBB, how this can modulate a propofol response and how bacterial traversal would be affected. We could demonstrate that bacterial traversal increases upon treatment of the cells with physiological doses of an AGE building compound. We also could show that anesthetics such as propofol and supplements in anesthesia including norepinephrine lead to an increase of the BBB permeability for bacteria. Importantly, the antioxidant ascorbic acid, more commonly known as vitamin C, can partly reverse the effects of the AGE building compound, propofol and norepinephrine. Similarly, it has been shown that ascorbic acid has positive effects on the permeability of glycated blood-brain barrier cells measured by transendothelial inulin transfer [9]. We propose that ascorbic acid can be beneficial in reducing the effects of diabetic encephalopathy which is accepted as an important complication of diabetes and preventing adverse effects on the BBB during anesthesia.

The BBB is a tight barrier separating brain parenchyma from circulating blood, whereas building of AGE lead to an increase of microbial traversal through human brain microvascular endothelial cells.

## 2. Experimental Section

### 2.1. Cells and Cell Culture

Transfected human brain microvascular endothelial cells (THBMEC) [34] were kindly provided by the group of MF Stins (Los Angeles, CA, USA). Cells were cultivated in DMEM F12 medium (Thermo Fisher Scientific, Waltham, MA, USA) at 37 °C in a humidified cell culture incubator. Supplemented medium was prepared by adding 100 mg/L penicillin and 100 mg/L streptomycin (Thermo Fisher Scientific), 2 mM L-glutamine (Thermo Fisher Scientific) and 4% heat-inactivated fetal calf serum (GE Healthcare, Little Chalfont, UK). Cells were passaged every 3–4 days. THBMECs were detached with Trypsin/EDTA (Thermo Fisher Scientific) and pelleted at 210 g for 3 min. Further particulars can be found in [33].

### 2.2. Treatment for Immunoblotting

For the immunoblots showing glycation of cells, THMBECs were incubated in medium supplemented with methylglyoxal (MGO) (Sigma Aldrich, St. Louis, MO, USA) for 1 h. Different concentrations, i.e., 0.05 mM, 0.15 mM, 0.45 mM, or 1 mM, of MGO were used for immunoblotting. Additionally, an immunoblot for detection of CD31 was performed. Therefore, cells were incubated with medium supplemented with interleukin 1β (IL1β) (Immunotools, Friesoythe, GER) and tumor necrosis factor α (TNFα) (Immunotools) for 24 h. A concentration of 0.05 ng/mL was used. Cells were incubated in a humidified incubator at 37 °C. Untreated THBMECs cultured in media served as a control.

### 2.3. Preparation of Cell Extracts

After incubation, treated and control cells were washed twice with PBS. Cells were removed from the surface by scraping and directly lysed into SDS-sample buffer (100 mL buffer containing 12.5% SDS, 0.3 M Tris, 50 mL glycerin, bromophenol blue at pH 6.8 and 1:10 DTT to buffer) which was pre-warmed for 5 min by 90 °C. After mixing, the cell extracts were used for downstream analysis.

### 2.4. Immunoblotting

Proteins of the samples were separated by SDS-PAGE on a 10% acrylamide gel and transferred afterwards to a nitrocellulose membrane for 1 h 15 min in blotting buffer with a constant amperage of 25 mA per gel. During the blotting process, the blot chamber (VWR, Radnor, PA, USA) was cooled down to avoid overheating. The following staining with ponceau red solution containing 0.1% ponceau S (Carl Roth, Karlsruhe, Germany), 3% trichloroacetic acid and 3% sulfosalicylic acid proved a successful protein transfer on to the membrane. The membrane was washed twice with water and was then blocked for 1 h at room temperature using 5% milk in TBS. The membrane was then incubated with the primary antibodies overnight at 4 °C. Glycation was detected by the monoclonal antibody CML-26 (ab12514, Abcam, Cambridge, CB2 0AX, UK) at a 1:10,000 dilution. CD31 was detected using the monoclonal anti-CD31-Antibody (ab24590, Abcam) at a dilution of 1:1000. VE-cadherin was detected by a monoclonal antibody (ab166715, Abcam), diluted 1:1000. Occludin was detected using anti-occludin-Antibody (ab167161, Abcam) at a 1:100,000 dilution. To detect integrin-β1, the membrane was blocked for 1 h at room temperature using 5% bovine serum albumin (BSA) (Carl Roth) in TBS. Afterwards, the membrane was incubated with anti-integrin-β1-antibody (Cell Signaling Technology, Frankfurt a. M., Germany diluted 1:1000 in BSA. After incubation with primary antibody, the membrane was washed three times with 1 x PBS for 10 min and incubated with the corresponding peroxidase-conjugated secondary antibody. Therefore, a monoclonal mouse-antibody (ab6789, Abcam) diluted 1:10,000 or rabbit-antibody (ab6721, Abcam) diluted 1:20,000 were used. The proteins were detected using Luminata Forte Western HRP-Substrate (Merck Millipore, Billerica, MA, USA) and signals were visualized using the ChemiDoc MP Imaging System (BioRad, Hercules, CA, USA). Occurring bands were analyzed using the associated ImageLab software (BioRad). Ponceau S staining served as loading control and was used to normalize the band intensity of the Western blot. The total protein normalization was recommended by the software producer as a better alternative to housekeeping protein normalization.

### 2.5. MTT-Assay

MTT assays were performed to determine the cytotoxicity of different cell treatments by measuring the metabolic activity. THBMECs were seeded into 96-well microtiter plates at a density of 1 × 10^5^ per well and treated with 0.15 mM MGO for 1 h, 3 µg/mL propofol (propofol 2%, Fresenius Kabi, SGP) for 3 h or 1 ng/mL norepinephrine (Arterenol^®^ 1 mg/mL, Sanofi-Aventis, Frankfurt a.M., GER) for 1 h added to medium. After treatment cells were washed with 200 µL PBS per well. MTT (Sigma, Saint Louis, MO, USA) was diluted to a concentration of 0.5 mg/mL in normal medium and 100 µL were added to each well and incubated for 4 h in a humidified incubator. After removal of MTT containing medium, remaining formazan crystals were dissolved in 150 µL DMSO (Sigma, Saint Louis, MO, USA). The absorbance was measured photometrically at a wavelength of 570 nm (background 630 nm) on a microplate reader Multiskan EX (Thermo Fisher Scientific, Rockford, IL, USA). Relative cell viability was calculated and compared to the untreated control, which were set to 100% of metabolic activity.

### 2.6. Measurement of Bacterial Traversal through the BBB

To test the effect of different compounds on the permeability of the human BBB, we developed an endothelial cell culture model, which mimics a tight BBB model with THBMECs. Cells were treated and exposed to bacteria as previously described [33]. In brief, THBMECs were grown on 12-well filters with 3.0-µm pore size (ThinCerts, Greiner Bio-One, Austria) to a confluent layer. Before seeding cells, filters were coated with 10 µg/mL collagen IV and 10 µg/mL fibronectin (Sigma, Saint Louis, MO, USA) mixture for 24 h. The cells were incubated for 14 days in a cell culture incubator with 5% CO_2_ atmosphere at 37 °C, changing the DMEM/F12 medium every 2 to 3 days in the upper and lower chamber. Afterwards, they were treated with the different compounds. In the next step, medium from both chambers was changed to antibiotic free DMEM/F12 medium and *E. coli* strain GM2163 (Fermentas Life Sciences, Lithuania) bacteria were then fed into the upper chamber. After 6 h, samples from the lower chamber were plated and colonies were counted.

### 2.7. Treatment for Measurement of Bacterial Traversal through the BBB

To treat the cell monolayer in our model, THBMECs were grown for 14 days to confluence which were proofed by evaluating cell density on the filters with a microscope. Exemplarily, we measured the transendothelial electrical resistance (TEER) of 70 Ω·cm^2^ after growing on filters for 14 days (Supplementary Figure S1). Afterwards, THBMECS were incubated in medium supplemented with different compounds. To test the effect of glycation, cells were washed with PBS and medium supplemented with 0.15 mM MGO was given into the upper and lower chamber for 1 h. To test the effect of anesthetics, cells were washed with PBS and medium where 3 µg/mL propofol for 3 h or 1 ng/mL norepinephrine were given into both chambers for 1 h. Incubation in medium supplemented with Intralipid (SMO Flipid 200 mg/mL, Fresenius Kabi) for 3 h and sodium metabisulfite for 1 h (PanReacAppliChem, ITW Reagents, Darmstadt, GER) served as control. To test the effect of vitamin C, cells were incubated with 0.1 mM ascorbic acid (AA) (Carl Roth) for 4 h added to medium in both chambers. Additionally, cells were first incubated with medium supplemented with 0.15 mM MGO and afterwards they were washed with PBS and medium supplemented with 0.1 mM AA were added. In addition, the reverse procedure was performed with AA treatment first followed by treatment with MGO. Untreated cells served as control.

### 2.8. RNA Extraction and Barrier Genes High-Throughput Multiplex qPCR

RNA was isolated using the NucleoSpinTM RNA kit according to manufacturer’s protocol (Macherey-Nagel, Cat. No. 740955.250) from the indicated cell lines. Samples of 250 ng RNA were transcribed to cDNA using the High-Capacity cDNA Reverse 191 Transcription Kit (Applied Biosystems, Cat. No. 4368814), according to the manufacturer’s instructions. Targets were preamplified using tenfold concentrated primer pools, mastermix, and the following program: 15 min at 95 °C for HotStar Plus Taq Polymerase (Qiagen, Cat. No. 203603), 18 cycles of 40 s at 95 °C, 40 s at 60 °C, 80 s at 72 °C, and 7 min at 72 °C. We used a BiomarkTM system containing an IFC Controller HX and 96.96 Dynamic ArraysTM IFC, according to the manufacturer’s instructions. In brief, each sample well was loaded with TagmanTM gene expression mastermix, DNA binding dye sample loading reagent, EvaGreenTM binding dye, and 1:8 diluted preamplified cDNA. Target wells were loaded with assay loading reagent and the respective primers. After qPCR and data allocation, the Ct values of the targets were normalized to the endogenous control B2M. The ΔCt values were used for the following statistical analysis applying the software Graph Pad Prism 7 (GraphPad, San Diego, CA, USA).

### 2.9. Statistical Analysis

Data are represented as bar diagrams or data points including mean ± standard error of measurement (SEM). Statistical analyses were performed using OriginPro2017 software (OriginLab Corporation, Northampton, MA, USA) or Graph Pad Prism 7 (GraphPad, San Diego, CA, USA). Unpaired student t-test against the control group or a theoretical value of 1 (due to data normalization) was used for microbial traversal after treatment with MGO. ANOVA and Tukey Kramer as post hoc tests were used for every other data. A difference between untreated and treated samples at *p* < 0.05 was considered as statistically significant, and significant *p*-values are displayed on each graph. RNA extraction and barrier genes high-throughput multiplex qPCR has been performed in two technical replicates for each cell line.

## 3. Results

In order to analyze the effects of glycation and anesthetics on bacterial traversal through the BBB, a model, which mimics the BBB, was built-up by human brain endothelial cells (THBMEC) [33,34]. These cells were grown on filters until confluence to prevent bacteria in the upper chamber from crossing into the lower chamber. Since this barrier is built-up by cell junctions of THBMECs, we firstly characterized the expression of common tight and adherens junction proteins at the beginning and end of the two weeks of cell cultivation. 

### 3.1. Determination of Protein Levels Building the Cell Junctions

**Figure 1:**
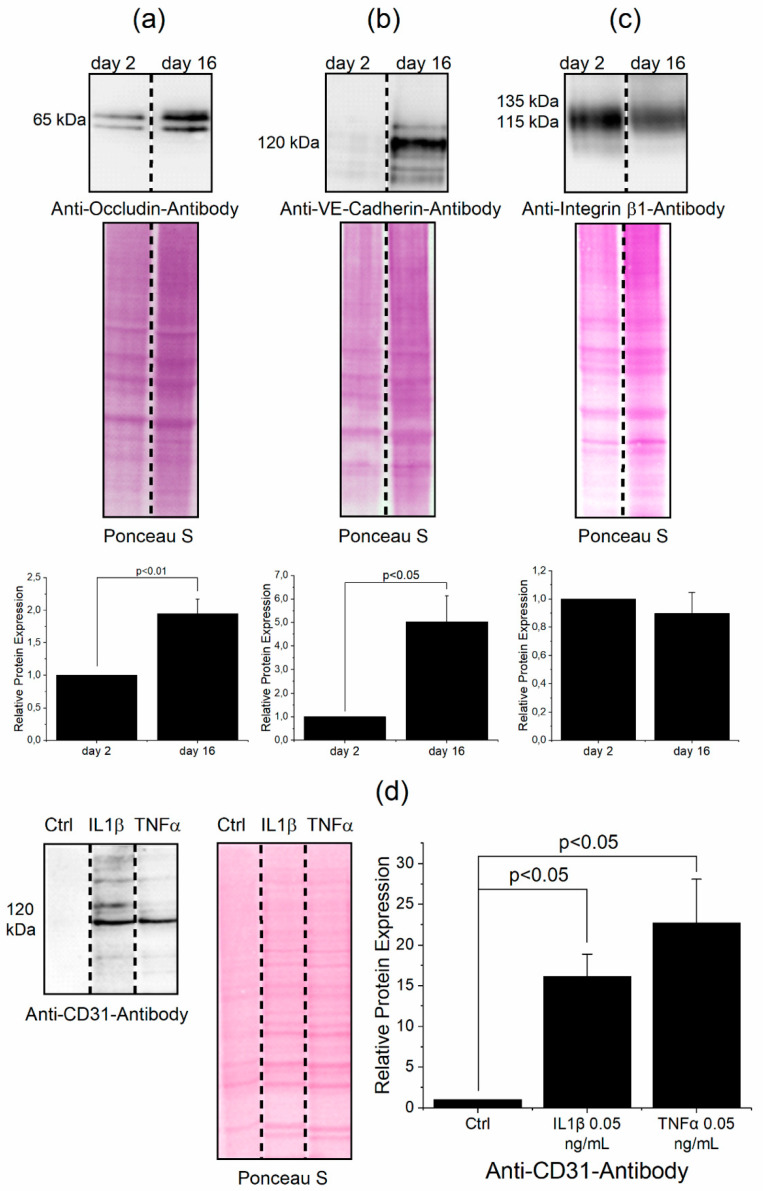
Determination of protein levels building the cell junctions in THMBECs. THBMECs were cultivated for 2 and 16 days. Afterwards, total protein was isolated and separated using SDS-PAGE. Expression of proteins was detected via immuno-blotting using anti-occludin antibody (**a**), anti-VE-cadherin antibody (**b**) and anti-integrin β1 antibody (**c**), respectively (*n* = 3). THBMECs were incubated with IL1β and TNFα at a concentration of 0.05 ng/mL for 24 h. Total protein was isolated and separated using SDS-PAGE. Expression of CD31 was detected by immuno-blotting using anti-CD31 antibody (**d**), (*n* = 3).

Occludin is an important protein of tight junctions, therefore we decided to analyze their levels by immunoblotting. There was a significant increase of occludin expression between day 2 and 16 (Figure 1a). VE-cadherin as a representative protein of adherens junctions increased between day 2 and 16 as well (Figure 1b). Integrin-β1 did not display increased expression over time (Figure 1c), but was also an important protein for cell adherence and cell signaling. We also treated the cells for 24 h with interleukin-1-beta (IL1β) and tumor necrosis factor alpha (TNFα) to analyze, whether THBMECs were able to react on proinflammatory mediators. A marker for this event was the platelet endothelial cell adherence molecule (PECAM or CD31), which primarily regulates leukocyte transmigration. The expression of CD31 increased regardless of whether using concentrations of 0.05 ng/mL or 10 ng/mL of IL1β or TNFα (Figure 1d), as proven by immunoblotting. The presence of mRNA coding for various other proteins with crucial functions in building tight junctions could be confirmed by qPCR in the THBMECs compared to hCMECs/D3 cells (Supplementary Figure S2).

### 3.2. Protein Glycation Is Induced by MGO

**Figure 2:**
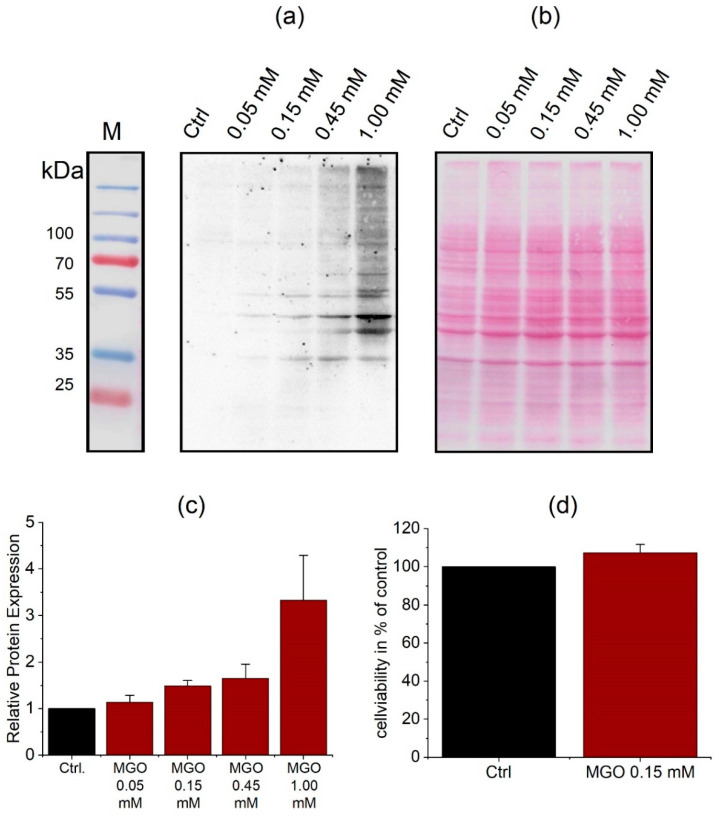
THBMECs were incubated with MGO at different concentrations for 1 h in normal medium. Total protein was isolated and separated using SDS-PAGE. Glycation of proteins was detected by immuno-blotting using anti-AGE-antibody (CML-26) (**a**). Ponceau S staining served as loading control (**b**). The bar graph shows the mean + SEM of relative protein glycation detected by immunoblotting, untreated cells serve as control (**c**), (*n* = 5). Cell viability in THBMECs upon treatment with MGO (**d**). Cells were treated with 0.15 mM MGO for 1 h. Afterwards, MTT assays were performed. The graph shows the mean + SEM of the absorbance of formazan crystals at a wavelength of 570 nm, untreated cells served as control, (*n* = 3).

To analyze the effect of glycation on cells, we treated them with MGO to induce formation of AGE. The treatment lasted 1 h and different concentrations of MGO were used to find the lowest AGE inducing concentration for further experiments. AGE were detected by immunoblotting after treatment with 0.05 mM, 0.15 mM, 0.45 mM, or 1 mM MGO (Figure 2a). The immunoblots were quantitated (Figure 2b). According to the results, we decided to use 0.15 mM MGO for 1 h in all our further experiments, since these concentrations have been measured in patients [35]. Additionally, cell viability assays were performed to assess the treatment of cells with 0.15 mM MGO for 1 h. The assay did not show toxic effects of 0.15 mM MGO treatment for 1 h on THBMECs or interference with cellular proliferation (Figure 2d). Untreated cells served as control in this assay. We also tested whether the expression levels of RAGE change during the treatment and whether the Western blot signal would be masked by AGE. We could detect RAGE in the THBMECs by immunoblotting with or without treatment of MGO, whereby the protein level did not change after an hour of treatment with MGO (Supplementary Figure S3).

### 3.3. Protein Glycation Increases the Permeability of the BBB, Which Can Be Reverted by Antioxidant

**Figure 3:**
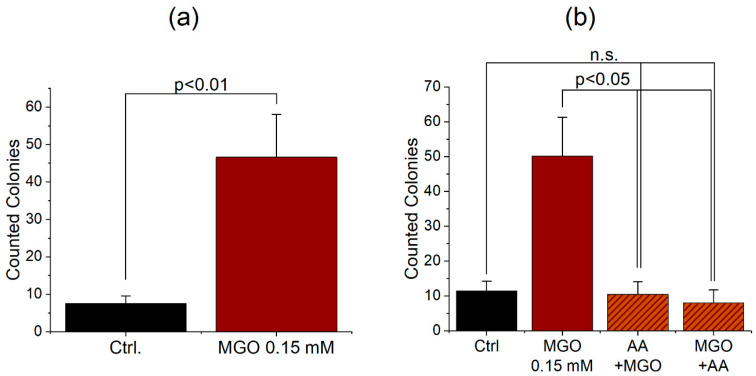
Increased microbial traversal through a BBB model upon treatment with MGO (**a**). THBMECs were treated with 0.15 mM MGO for 1 h. 450 µL of bacteria suspension (OD 0.5) were put into each upper chamber. Medium from the lower chamber was plated on agar plates after 6 h. Graphs show the mean + SEM of counted colonies, untreated cells serve as control, (*n* = 3). Microbial traversal in the presence of ascorbic acid decreased in MGO-treated cells (**b**). THBMECs were treated with 0.15 mM MGO for 1 h. Afterwards, ascorbic acid with a concentration of 0.1 mM was administered to the treated cells. Additionally, cells were first treated with ascorbic acid for 4 h and afterwards glycated with MGO 0.15 mM for 1 h. 450 µL of *E. coli* suspension (OD 0.5) was put into each upper chamber. Medium from the lower chamber was plated on agar plates after 6 h. Graphs show the mean + SEM of counted colonies, untreated cells served as control, (*n* = 3).

In order to analyze the effect of glycation on bacterial traversal, we built the model of human BBB with THBMECs and after cultivation of 14 days, cells were treated with 0.15 mM MGO for 1 h. The upper chamber was then inoculated with bacteria and, after 6 h, medium from the lower chamber was plated and colonies were counted. Untreated cells served as control and showed a low bacterial traversal with 6.2 colonies on average. Compared to this 0.15 mM MGO for 1 h affected the traversal by increasing the number of colonies up to 47 colonies on average (Figure 3). In addition, we wanted to test some compounds with anti-glycation effects in our model of the human blood-brain barrier. An agent we took into consideration fulfilling the requirement was ascorbic acid (AA), better known as vitamin C. It is a reducing agent, but in addition, it provided the risk of glycation by itself [36]. We used 0.1 mM ascorbic acid for 4 h and MGO with 0.15 mM for 1 h to treat cells after 14 d of cultivation. Additionally, we combined both treatments—first ascorbic acid then MGO or vice versa. The glycated cells with the additional ascorbic acid treatment showed a significant decrease of microbial traversal compared to cells, which were only treated with MGO (Figure 3).

### 3.4. Anesthetics Increase the Permeability of the BBB and Bacterial Traversal

Anesthetics are known to have an impact on permeability of the human blood-brain barrier. Therefore, we decided to test common anesthetics in the human BBB model. Additionally, we combined glycation and anesthetic treatment to exemplary replicate the situation of an older or diabetic person during surgery. We used propofol (PP) as one of the most common anesthetic compounds and norepinephrine (NE), which is used to compensate for a decrease of blood pressure caused by propofol. Treatment of cells was performed with 3 µg/mL propofol [37,38] and 1 ng/mL norepinephrine [39]. These concentrations are in the range of common blood levels during surgery. The propofol treatment lasted 3 h to simulate an average surgery. Norepinephrine is mostly used as bolus injection, so we treated cells for 1 h in our model. 

**Figure 4:**
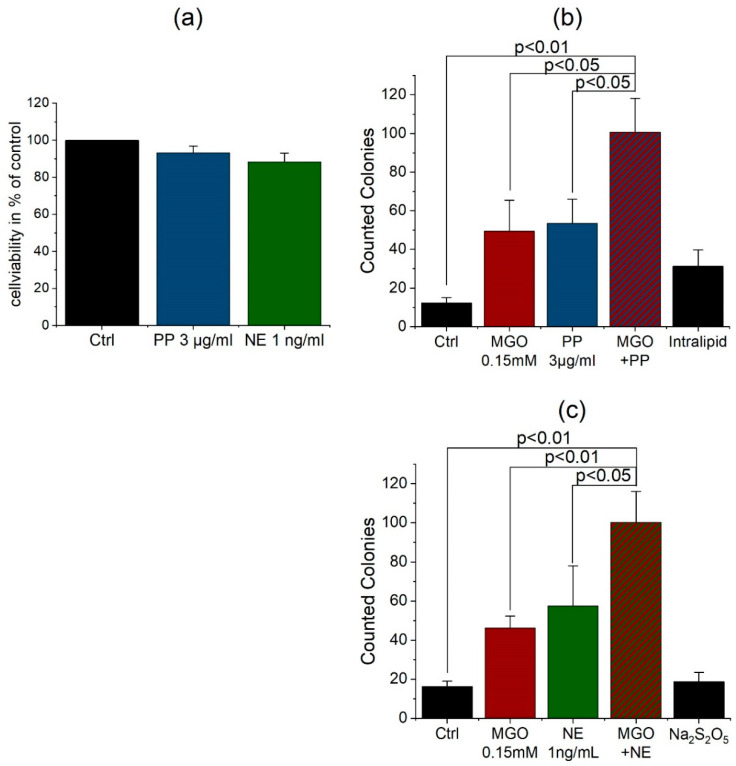
Cell viability of the compounds propofol (PP) and norepinephrine (NE) in endothelial cells (**a**). THBMECs were treated with 3 µg/mL propofol for 3 h and 1 ng/mL norepinephrine for 1 h. MTT assays were performed to analyze the cell viability after treatment. The graph shows the mean + SEM of the cell viability, untreated cells served as control, (*n* = 3). Microbial traversal in presence of propofol and norepinephrine with and without MGO treatment. THBMECs were treated with 0.15 mM MGO for 1 h. Afterwards, glycated and non-glycated cells were treated with 3 µg/mL propofol for 3 h (**b**) or with 1 ng/mL norepinephrine for 1 h (**c**). 450 µL of *E. coli* suspension (OD 0.5) were put into each upper chamber. Medium from the lower chamber was plated on agar plates after 6 h. The graph shows the mean + SEM of counted colonies, untreated cells and intralipid (**b**) or sodium metabisulfite (**c**) served as control, (*n* = 3).

Again, we performed cell viability assays for the treatment of cells with propofol 3 µg/mL for 3 h and norepinephrine 1 ng/mL for 1 h. The assay showed no toxic effect of propofol or norepinephrine treatment on THBMECs or interference with proliferation (Figure 4). Untreated cells served as control in this assay. According to the test results, we treated the THBMECs in our model after 14 d cultivation with 3 µg/mL propofol for 3 h or 1 ng/mL norepinephrine for 1 h. To see effects of AGE formation during surgery, we glycated cells first with 0.15 mM MGO for 1 h and treated them with propofol or norepinephrine. After treatment, the bacteria solution was put into the upper chamber and after 6 h, medium from the lower chamber was plated and colonies were counted. Untreated cells served as control. There was a significant increase of microbial traversal between non-glycated and glycated cells treated with propofol. As control, we used a soya oil solution (Intralipid), since common propofol compounds are mixed with soya oil. Additionally, we were able to show an increase of microbial traversal between non-glycated and glycated cells after norepinephrine treatment. Sodium metabisulfite served as control, because it is used for the preservation of norepinephrine. 

## 4. Discussion

Our results demonstrated the effect of glycation on the permeability of the human blood-brain barrier. As many studies proved a correlation of diabetes with meningitis [19,20,21] and post-operative delirium (POD) [18], we were able to show an effect of AGE on our model of the human BBB. A significant increase of microbial traversal in the model of the BBB in THBMECs was observed after glycation with MGO. We used a low amount of MGO which did not affect the cell viability as MTT assays (Figure 2d) indicate. This concentration is at the lower range of measured human serum concentrations of MGO (190 +/− 68 nmol/L) [35]. These results indicate an effect of AGE formation on the transcellular and intracellular barrier function of THBMECs, which is associated with paracellular or transcellular traversal of bacteria [22,23,24]. Furthermore, we measured the transendothelial electrical resistance (TEER) (Supplementary Figure S1) proving that a breakdown of the BBB is not causative for the resulting effects. A decrease of resistance after treatment with MGO, propofol, or norepinephrine could not be measured in our model. 

The treatment with propofol and norepinephrine was performed with amounts as similar as possible to plasma concentrations during surgery [37,38,39]. To show effects in diabetic patients, cells were glycated with MGO before inoculating with bacteria. The single treatment with propofol indicates a damaging effect and a support of the breakdown of BBB as some studies also proposed [29,30]. As the effects of propofol are distinct in our experiments, we cannot confirm neuroprotective effects of propofol as some studies comparing to sevoflurane anesthesia propose [32]. Single treatment with norepinephrine increases the permeability of BBB as well. In animal models, high levels of norepinephrine correlated with POD [40], whereas the circumstances in humans have not been entirely elucidated so far [41]. Whilst norepinephrine is important for maintaining hemodynamical stability in ICU patients, it led to an increase of the microbial traversal across the BBB in THBMECs. The results indicate an additive effect of AGE formation and propofol or norepinephrine on the permeability of human blood-brain barrier (Figure 4). The wide influence of AGE on proteins and the effect of RAGE seem to amplify the negative effect of anesthesia on the permeability of human BBB. As diabetes is associated with a high level of AGE, the results highlight the importance of spare anesthesia in diabetic patients and a well-adjusted blood glucose level before surgery [42].

Since ascorbic acid is a reducing agent [43], it is able to prevent glycation of proteins like hemoglobin [44]. Our results demonstrate that ascorbic acid partly reverses the effect of glycation on the permeability of the human BBB (Figure 3b). Patients after surgery tend to have a high amount of reactive oxygen species (ROS), which often exceed their antioxidant capacity [45]. This leads to damage of macromolecules and ends up in organ dysfunction including POD. Ascorbic acid is known to be one of the most important antioxidants to reduce the influence of free radicals [46] and systemic inflammatory reaction [47]. In addition to the systemic impact of ascorbic acid, our experiments show a positive effect on glycated cells. As hyperglycemia is associated with a high level of ROS [48], ascorbic acid prevents the formation of AGE [49]. Thus, our results indicate an effect of ascorbic acid after the formation of AGE. The human body depends on an adequate intake of vitamin C, as it is unable to synthesize ascorbic acid by itself. Given that 0.1 mM ascorbic acid has been used in the experiments, it reflects a realistic concentration, if 100 mg ascorbic acid are ingested in a person with a blood volume of 4–6 L on average. We propose that high levels of ascorbic acid in the body fluids may have beneficial effects of diabetic encephalopathy, which is accepted to be a major complication of diabetes mellitus and prevent adverse effects during anesthesia.

This model provides the possibility to test the influence of agents on the permeability of the human BBB, whereby the absolute measurement of bacteria traversal across the cell monolayer allows a fast testing to analyze further drugs.

Further research is needed to determine the mainly affected proteins. A screen via mass spectrometry could give first insights of altered protein levels and associated pathways, which could be confirmed with cell biological experiments. The route of the bacteria during the traversal from the apical side to the basolateral side is also not entirely clear. Thus, tracking experiments and monitoring of the bacteria by microscopy could help to elucidate their routes.

## 5. Conclusions

In summary, we could demonstrate an increase of microbial traversal across the human BBB after treatment with AGE as well as propofol and norepinephrine in our THBMEC model. Importantly, these results could be partially reversed upon the administration of ascorbic acid, which could have beneficial effects, if given prior to anesthesia.

## Supplementary Materials

The following are available online at https://www.mdpi.com/2077-0383/9/11/3672/s1, Figure S1: Measurement of Colony Building Units (CBU) at an optical density with a range from 4.5 to 5.5. at 570 nm (a). Transendothelial electrical resistance measured in the BBB model of THBMEC after different time points and treatments (b, c), Figure S2: Heat map analysis of high-throughput multiplex barrier qPCR data of THBMEC cells in comparison to hCMEC/D3 cells as positive control, set to 1.0. Total RNA extracts were pre-amplified and the Ct values of the targets were normalized to the endogenous control B2M after qPCR, Figure S3: Expression levels of RAGE in THBMECs with or without MGO treatment. THBMECs were incubated with 0.15 mM MGO for 1 h in serum-free medium. Total protein was separated using SDS-PAGE. Expression of RAGE was detected by immuno-blotting using anti-RAGE-antibody (ab3611) (a). Tubulin detected with an anti-tubulin antibody as well as Ponceau S staining served as loading control (b).
