# Supplementary material for: Glycation Increases the Risk of Microbial Traversal through an Endothelial Model of the Human Blood-Brain Barrier after Use of Anesthetics
Source: J Clin Med. 2020 Nov 16;9(11):3672. doi: 10.3390/jcm9113672 (PMC7698006; doi:10.3390/jcm9113672)
Supplement: Supplementary file 1 [file jcm-09-03672-s001.zip › jcm-976467supp/supple.docx]

**Supplementary Materials:**


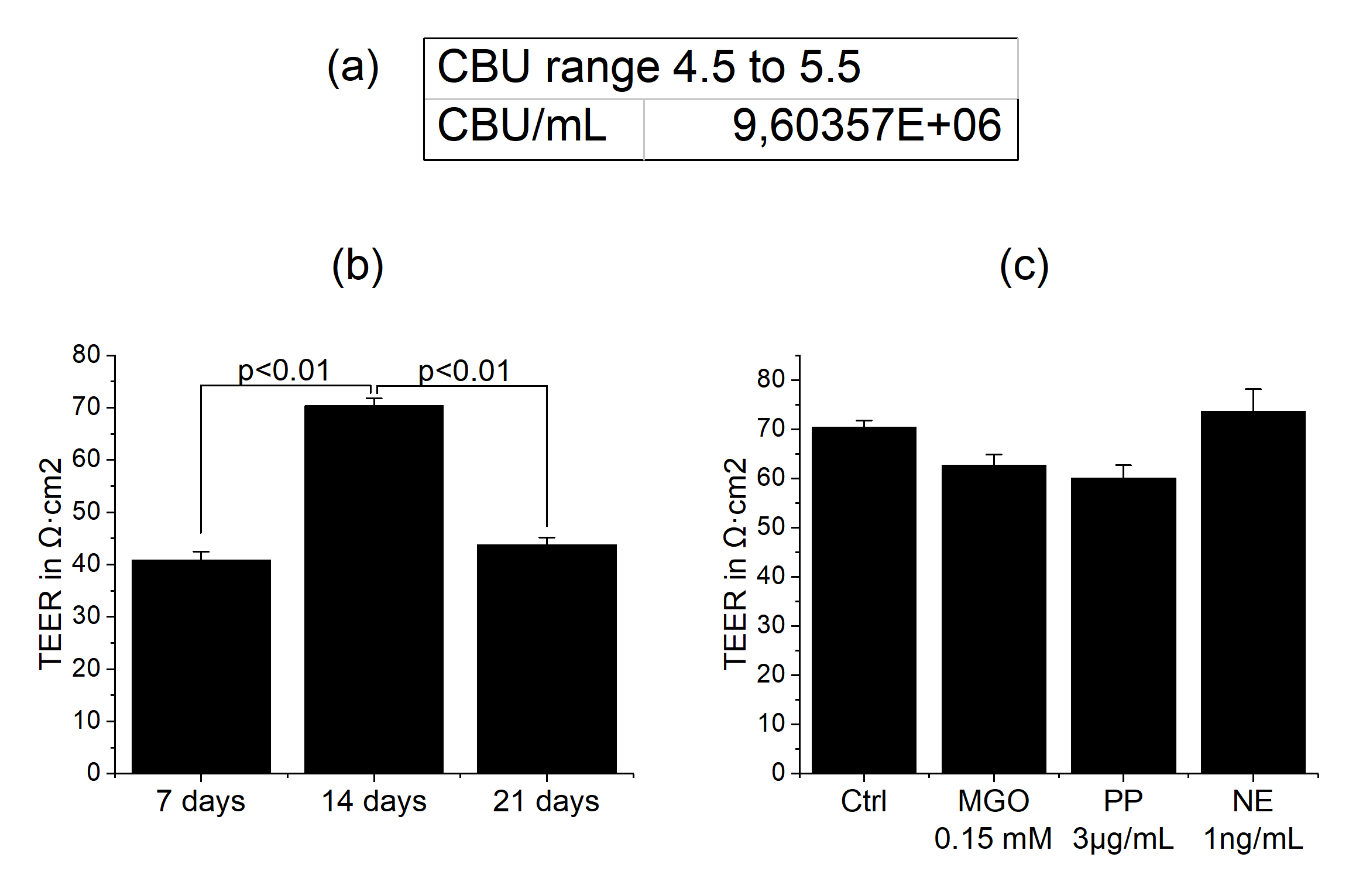


**Figure 1.** Measurement of Colony Building Units (CBU) at an optical density with a range from 4.5 to 5.5. at 570 nm (a). Transendothelial electrical resistance measured in the BBB model of THBMEC after different time points and treatments (b, c).


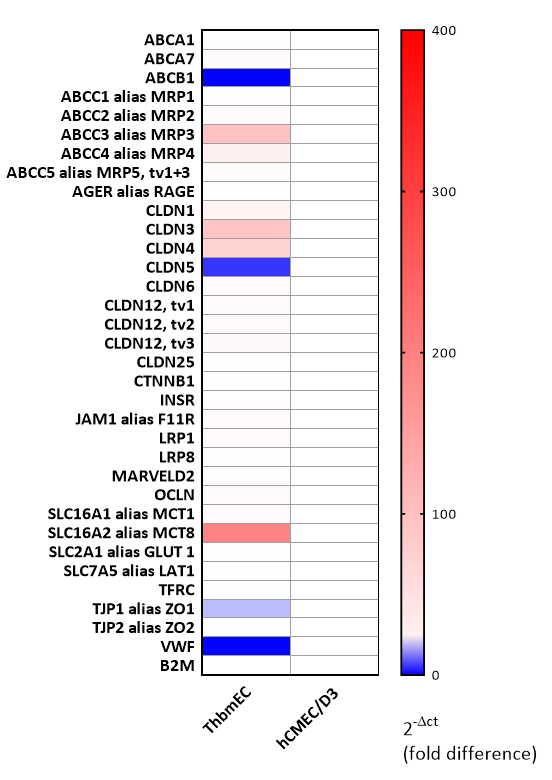


Figure S2: Heat map analysis of high-throughput multiplex barrier qPCR data of THBMEC cells in comparison to hCMEC/D3 cells as positive control, set to 1.0. Total RNA extracts were pre-amplified and the Ct values of the targets were normalized to the endogenous control B2M after qPCR.


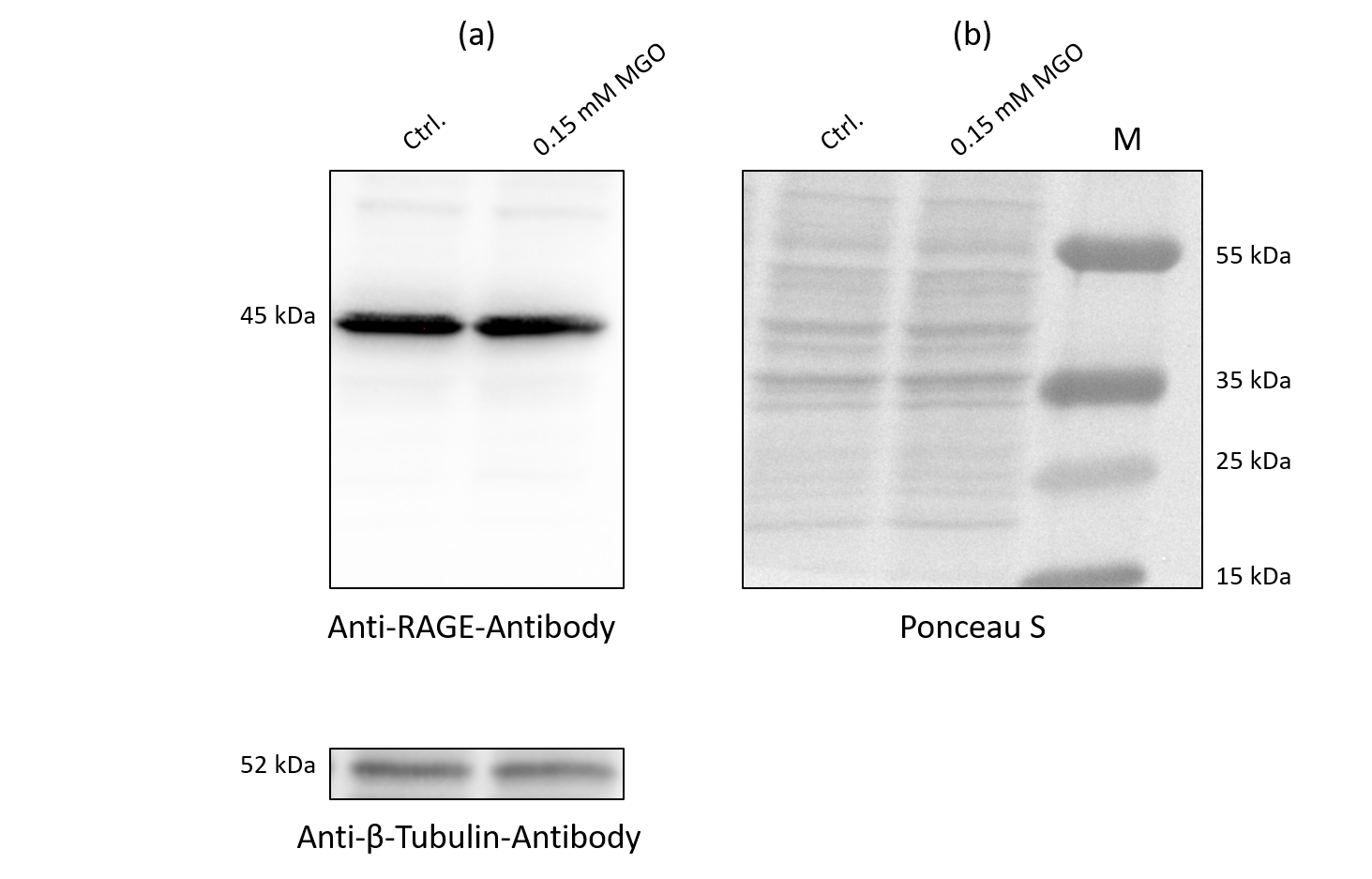


Figure S3: Expression levels of RAGE in THBMECs with or without MGO treatment. THBMECs were incubated with 0.15 mM MGO for 1 h in serum-free medium. Total protein was separated using SDS-PAGE. Expression of RAGE was detected by immuno-blotting using anti-RAGE-antibody (ab3611) (a). Tubulin detected with an anti-tubulin antibody as well as Ponceau S staining served as loading control (b).
