# Supplementary figures and images for: Glycation Increases the Risk of Microbial Traversal through an Endothelial Model of the Human Blood-Brain Barrier after Use of Anesthetics
Source: J Clin Med. 2020 Nov 16;9(11):3672. doi: 10.3390/jcm9113672 (PMC7698006; doi:10.3390/jcm9113672)

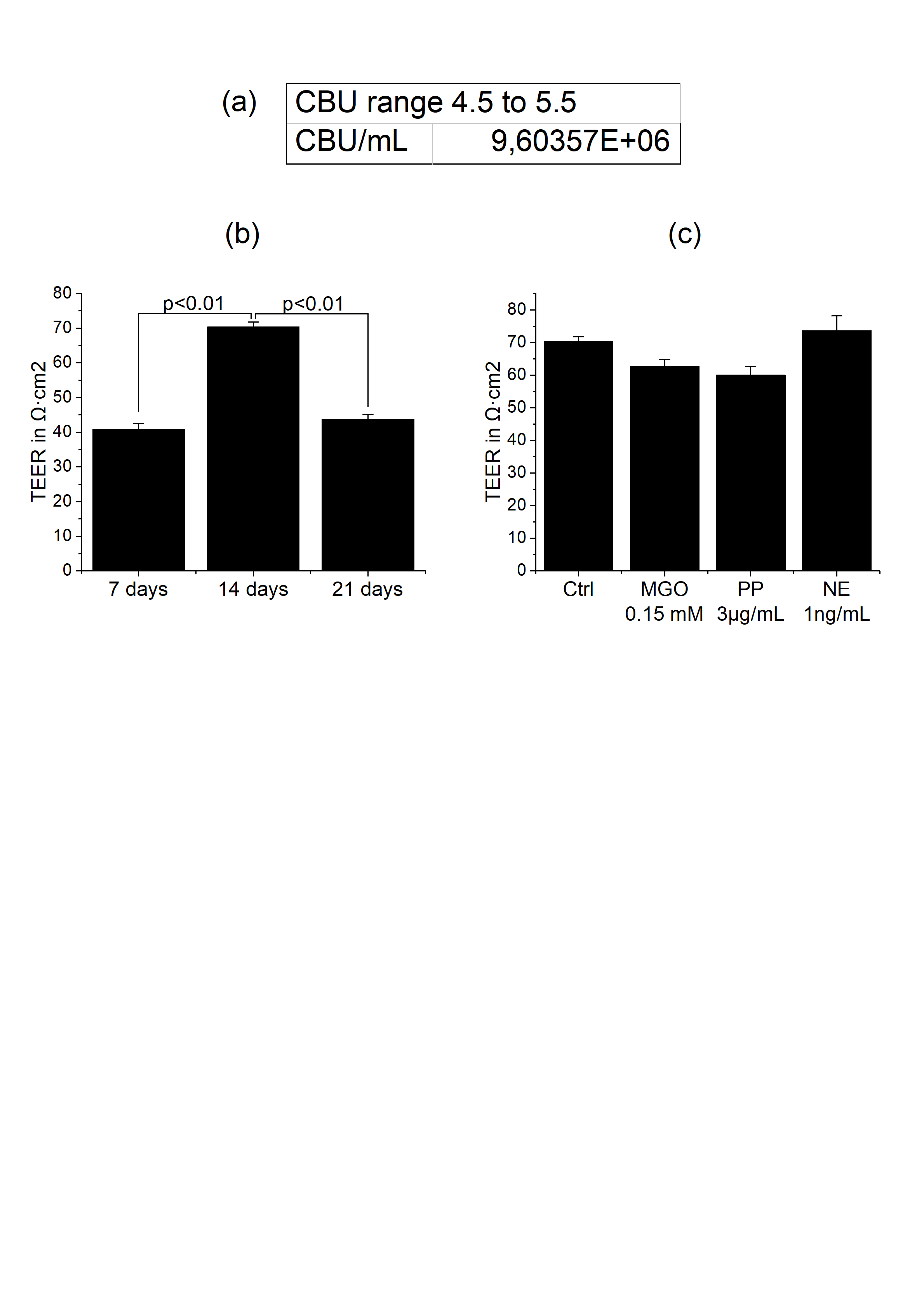

Supplement: Supplementary file 1 [file jcm-09-03672-s001.zip › jcm-976467supp/Supplementary figures/Figure S1.png]

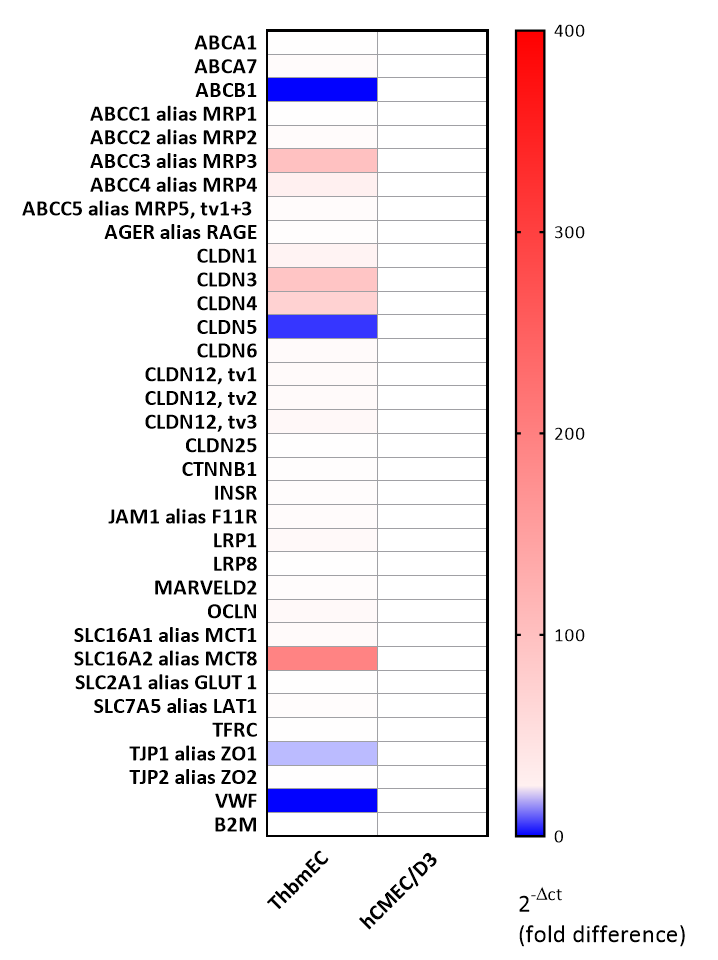

Supplement: Supplementary file 1 [file jcm-09-03672-s001.zip › jcm-976467supp/Supplementary figures/Figure S2.png]

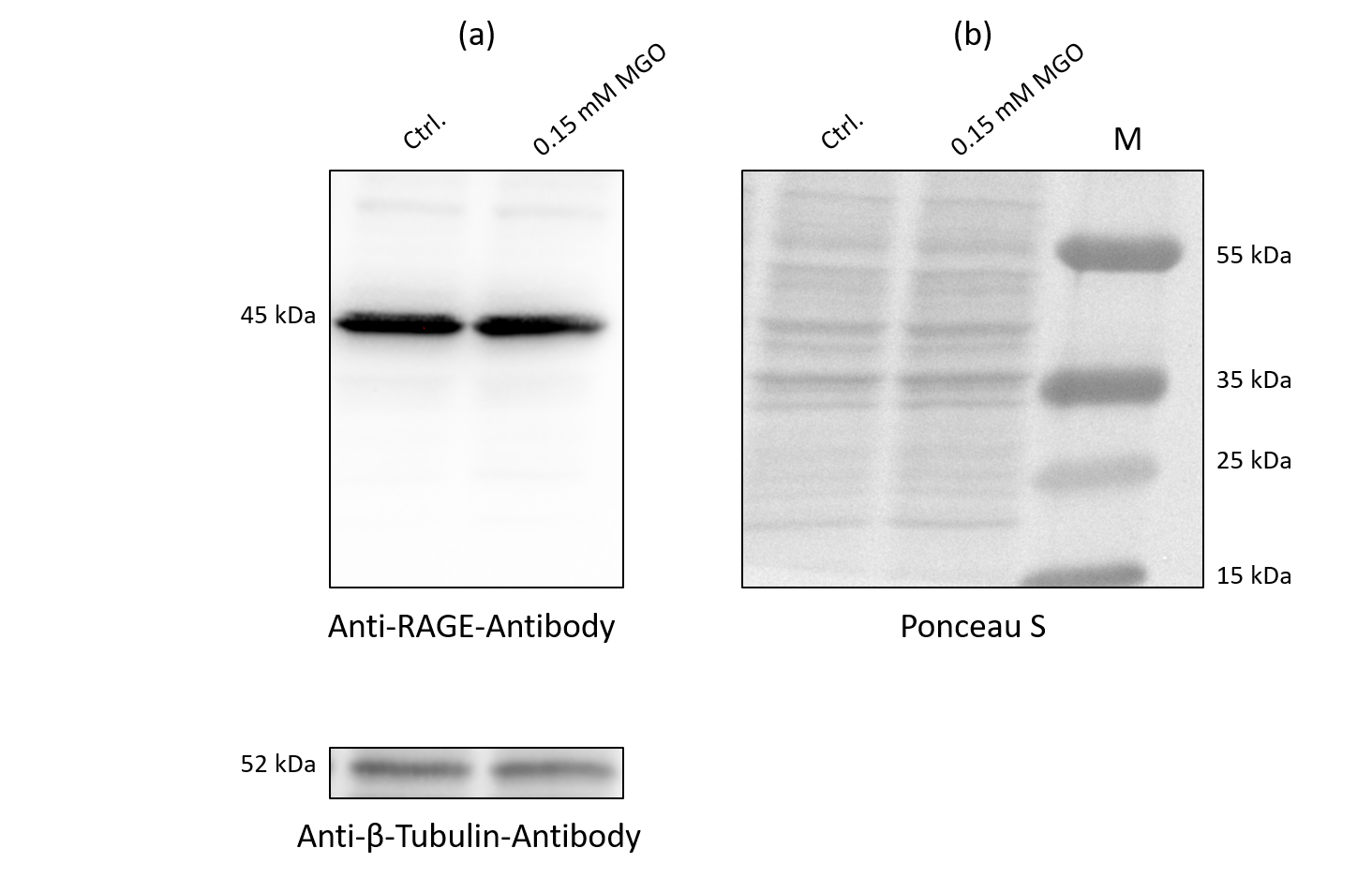

Supplement: Supplementary file 1 [file jcm-09-03672-s001.zip › jcm-976467supp/Supplementary figures/Figure S3.png]
